# An Indel Polymorphism in the *MtnA* 3' Untranslated Region Is Associated with Gene Expression Variation and Local Adaptation in *Drosophila melanogaster*

**DOI:** 10.1371/journal.pgen.1005987

**Published:** 2016-04-27

**Authors:** Ana Catalán, Amanda Glaser-Schmitt, Eliza Argyridou, Pablo Duchen, John Parsch

**Affiliations:** 1 Faculty of Biology, Ludwig-Maximilians-Universität München, Planegg, Germany; 2 Department of Ecology and Evolutionary Biology, University of California, Irvine, Irvine, California, United States of America; 3 Department of Biology and Biochemistry, University of Fribourg, Fribourg, Switzerland; University of California Davis, UNITED STATES

## Abstract

Insertions and deletions (indels) are a major source of genetic variation within species and may result in functional changes to coding or regulatory sequences. In this study we report that an indel polymorphism in the 3’ untranslated region (UTR) of the metallothionein gene *MtnA* is associated with gene expression variation in natural populations of *Drosophila melanogaster*. A derived allele of *MtnA* with a 49-bp deletion in the 3' UTR segregates at high frequency in populations outside of sub-Saharan Africa. The frequency of the deletion increases with latitude across multiple continents and approaches 100% in northern Europe. Flies with the deletion have more than 4-fold higher *MtnA* expression than flies with the ancestral sequence. Using reporter gene constructs in transgenic flies, we show that the 3' UTR deletion significantly contributes to the observed expression difference. Population genetic analyses uncovered signatures of a selective sweep in the *MtnA* region within populations from northern Europe. We also find that the 3’ UTR deletion is associated with increased oxidative stress tolerance. These results suggest that the 3' UTR deletion has been a target of selection for its ability to confer increased levels of *MtnA* expression in northern European populations, likely due to a local adaptive advantage of increased oxidative stress tolerance.

## Author Summary

Although molecular variation is abundant in natural populations, understanding how this variation affects organismal phenotypes that are subject to natural selection remains a major challenge in the field of evolutionary genetics. Here we show that a deletion mutation in a noncoding region of the *Drosophila melanogaster Metallothionein A* gene leads to a significant increase in gene expression and increases survival under oxidative stress. The deletion is in high frequency in three distinct geographic regions: in northern European populations, in northern populations along the east coast of North America, and in southern populations along the east coast of Australia. In northern European populations the deletion shows population genetic signatures of recent positive selection. Thus, we provide evidence for a regulatory polymorphism that underlies local adaptation in natural populations.

## Introduction

Natural populations adapt constantly to their changing environments, with alterations in protein sequences and gene expression providing the main sources of variation upon which natural selection can act. At present, understanding how changes in gene expression contribute to adaptation is one of the major challenges in evolutionary genetics. The fruit fly *Drosophila melanogaster* has populations distributed throughout the world, with environments ranging from tropical to temperate. On the basis of biogeographical, anatomical and population genetic studies, the center of origin of *D*. *melanogaster* has been inferred to be in sub-Saharan Africa [1–3]. Several genomic studies concluded that *D*. *melanogaster* underwent a population expansion around 60,000 years ago within Africa that set the ground for an out-of-Africa expansion 13,000–19,000 years ago and the subsequent colonization of Europe and Asia 2,000–5,000 years ago [4–6]. Because the colonization of new habitats requires that species adapt to new environmental conditions, there has been considerable interest in identifying the genetic and phenotypic changes that occurred during the out-of-Africa expansion of *D*. *melanogaster* [7–9].

**Fig 1:**
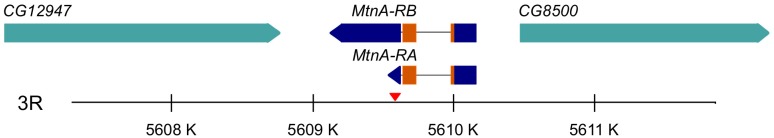
Structure of the *MtnA* locus. Two transcripts that differ only in their 3’ UTRs have been annotated for *MtnA* (*MtnA-RA* and *MtnA-RB*). Dark blue boxes represent the UTRs with the arrowheads indicating the direction of transcription. Orange boxes represent the coding exons. The thin lines joining the coding exons represent introns. The location of the polymorphic indel, which is shared by both transcripts, is indicated by the red triangle. For the coding genes flanking *MtnA* (CG12947 and CG8500), only the whole gene model is shown.

In order to identify genes that differed in expression between a *D*. *melanogaster* population from Europe (the Netherlands) and one from sub-Saharan Africa (Zimbabwe), whole-transcriptome comparisons were carried out using adult males and females [10,11], as well as the dissected brains and Malpighian tubules of each sex [12,13]. These studies identified several hundred genes that were differentially expressed between the two populations and which represent candidates for adaptive regulatory evolution. One of the candidate genes that showed a large difference in expression between populations in the brains of both sexes was the metallothionein (MT) gene *Metallothionein A* (*MtnA*). *MtnA* lies on chromosome arm 3R (Fig 1) and belongs to a gene family of five members that also includes *MtnB*, *MtnC*, *MtnD* and *MtnE* [14,15]. Metallothioneins are present in all eukaryotes and have also been identified in some prokaryotes [16]. In general, MTs are cysteine-rich proteins, a feature that makes them thermostable, and have a strong affinity to metal ions, especially zinc and copper ions [17]. Some of the biological functions that have been described for MTs include: sequestration and dispersion of metal ions; zinc and copper homeostasis; regulation of the biosynthesis of zinc metalloproteins, enzymes and zinc dependent transcription factors; and protection against reactive oxygen species, ionizing radiation and metals [18]. In natural isolates of *D*. *melanogaster*, increased *MtnA* expression has been linked to copy number and insertion and deletion (indel) variation and is associated with increased tolerance to heavy metals [19,20].

In this paper we show that the expression difference of *MtnA* between a European and a sub-Saharan African population is not associated with copy number variation, but is associated with a derived 49-bp deletion in the *MtnA* 3’ untranslated region (UTR). Outside of sub-Saharan Africa, the deletion shows a latitudinal cline in frequency across multiple continents, reaching very high frequencies in northern Europe. Using transgenic reporter genes, we show that the indel polymorphism in the 3’ UTR contributes to the expression difference observed between populations. Furthermore, we use hydrogen peroxide tolerance assays to show that the deletion is associated with increased oxidative stress tolerance. Population genetic analyses indicate that *MtnA* has been the target of positive selection in non-African populations. Taken together, these results suggest that a *cis*-regulatory polymorphism in the *MtnA* 3’ UTR has undergone recent positive selection to increase *MtnA* expression and oxidative stress tolerance in derived northern populations of *D*. *melanogaster*.

## Results

### Differential expression of *MtnA* between an African and a European population of *D*. *melanogaster*

**Fig 2:**
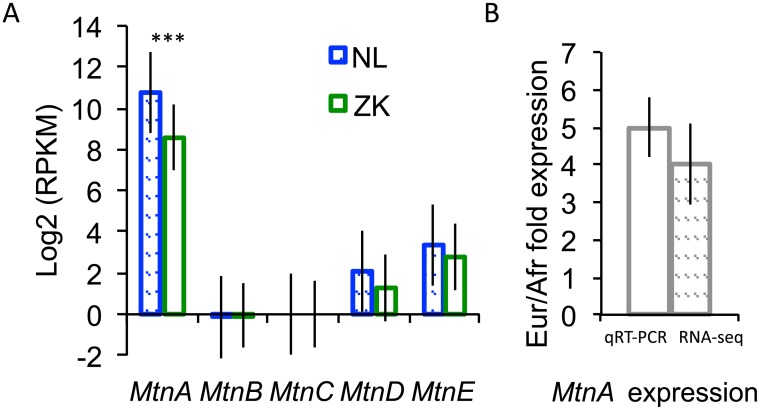
Expression of metallothionein genes in the brain in two populations of *D*. *melanogaster*. (A) Expression level of *Mtn* paralogs in the brain from RNA-seq data. Expression is reported in reads per kilobase per million mapped reads (RPKM). Only *MtnA* showed a significant difference in expression between a European (the Netherlands), shown in blue, and an African (Zimbabwe), shown in green, population (adjusted *P* < 10^−7^ in the RNA-seq analysis [12]). Expression of *MtnC* was not detected. (B) *MtnA* expression in the brains of European and African flies, as determined by qRT-PCR. The expression difference between populations is highly significant (*t*-test, *P* = 5x10^-5^). In both panels, the error bars indicate the standard error of the mean.

A previous RNA-seq study of gene expression in the brain found *MtnA* to have four times higher expression in a European population (the Netherlands) than in a sub-Saharan African population (Zimbabwe) [12]. Of the members of the *Mtn* gene family, only *MtnA* showed high levels of expression and a significant difference in expression between populations (Fig 2A). To confirm this expression difference, we performed qRT-PCR on RNA extracted from dissected brains of flies from each population following the same pooling strategy used previously [12]. With this approach, we found *MtnA* to have 5-fold higher expression in the European population than in the African population (Fig 2B).

The RNA-seq and qRT-PCR analyses were performed on a "per gene" basis and did not discriminate between the two annotated transcripts of *MtnA*, which differ only in the length of their 3' UTR (Fig 1). The *MtnA-RA* transcript completely overlaps with that of *MtnA-RB* and contains no unique sequence. The *MtnA-RB* transcript, however, contains an extra 371 bp at the 3' end that can be used to assess isoform-specific expression. Using RNA-seq data [12], we found that the *MtnA-RB* isoform represents only a small proportion of total *MtnA* expression (1.50% in the European population and 0.13% in the African population). Thus, almost all of the observed expression difference in *MtnA* can be attributed to the *MtnA-RA* isoform. Although present at very low levels, the *MtnA-RB* transcript showed much higher expression (50-fold) in Europe than in Africa (S1 Table).

### Absence of *MtnA* copy number variation

**Fig 3:**
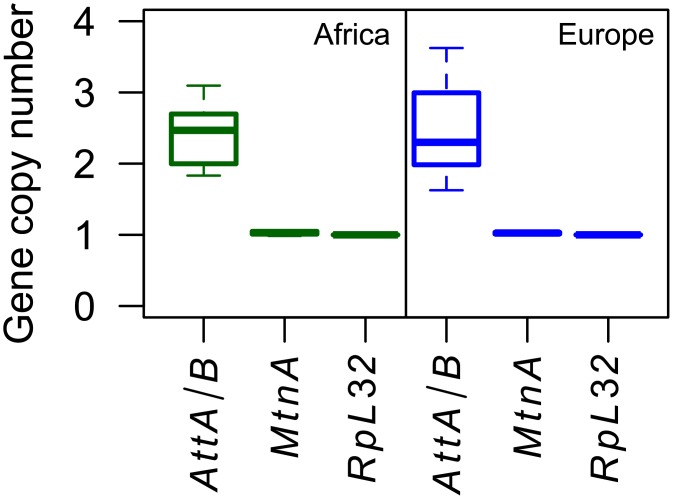
Results of CNV assays. Flies from Africa (Zimbabwe), shown in green, and Europe (the Netherlands), shown in blue, were tested for *MtnA* CNV. The close paralogs *AttA* and *AttB* were used as a positive control for multiple gene copies, while *RpL32* was used as a single-copy reference.

Previous studies found copy number variation (CNV) for *MtnA* in natural isolates of *D*. *melanogaster* and showed that an increase in copy number was associated with higher *MtnA* expression [19,20]. To determine if CNV could explain the observed expression difference between the European and the African populations, we assayed *MtnA* copy number in flies of both populations by quantitative PCR. We found no evidence for CNV within or between the populations (Fig 3). In both populations, *MtnA* copy number was equal to that of the control single-copy gene *RpL32* and was about half that of the nearly-identical paralogs *AttA* and *AttB* [21], which can be co-amplified by the same PCR primers and serve as a positive control. These results indicate that CNV cannot account for the observed variation in *MtnA* gene expression.

### An indel polymorphism in the *MtnA* 3' UTR is associated with expression variation

**Fig 4:**
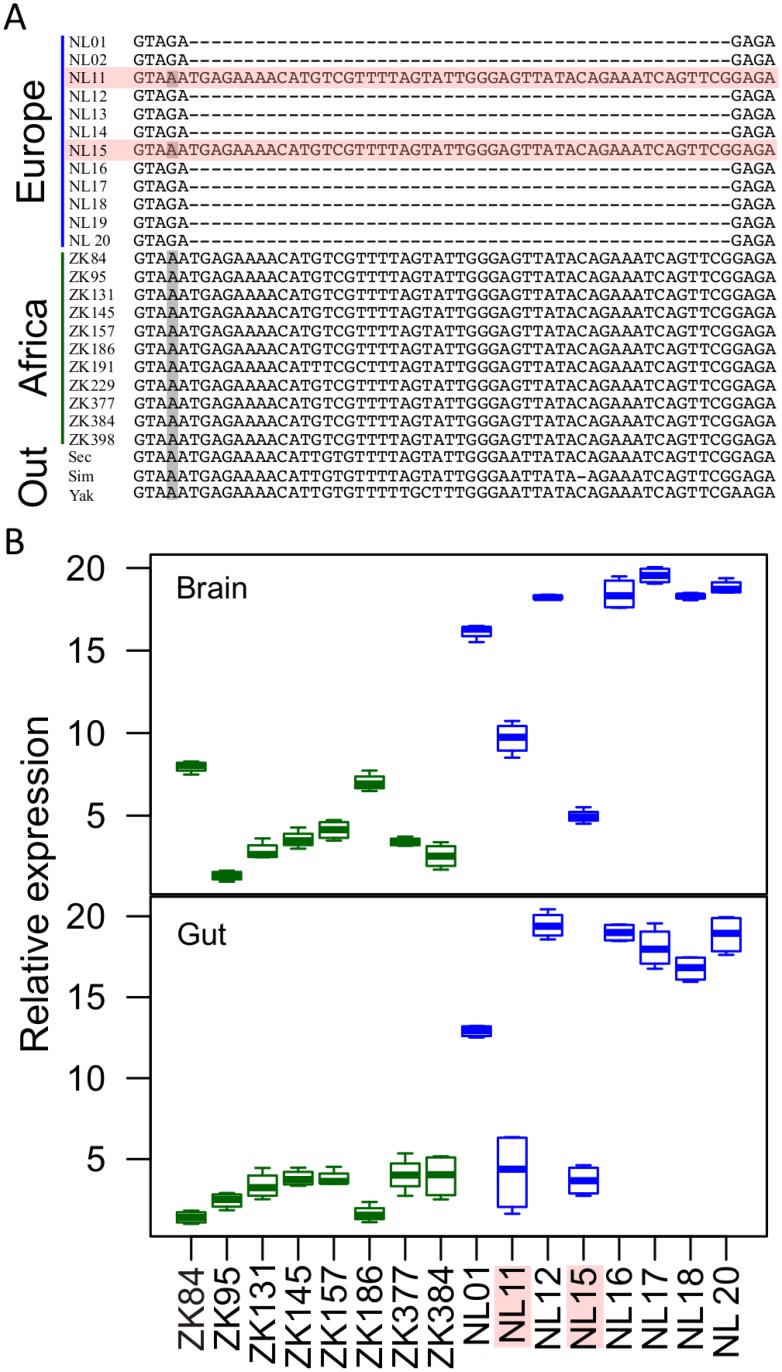
Association between an indel polymorphism in the *MtnA* 3' UTR and gene expression variation. (A) An indel (and a linked SNP marked in gray) in the *MtnA* 3' UTR are the only polymorphisms within the 6-kb *MtnA* region that show a large difference in frequency between an African and a European population of *D*. *melanogaster*. A comparison with three outgroup species, *D*. *sechellia* (Sec), *D*. *simulans* (Sim) and *D*. *yakuba* (Yak), indicated that the deletion is the derived variant. (B) *MtnA* expression in the brain and the gut of eight European (NL) lines, shown in blue, and eight African (ZK) lines, shown in green. The two European lines lacking the deletion, *NL11* and *NL15*, show lower *MtnA* expression than those with the deletion.

To identify *cis*-regulatory variants that might be responsible for the difference in *MtnA* expression between European and African flies, we sequenced a 6-kb region encompassing the *MtnA* transcriptional unit (Fig 1) in 12 lines from the Netherlands (NL) and 11 lines from Zimbabwe (ZK). In addition, we quantified *MtnA* expression in a subset of eight lines from each population in both the brain and the gut by qRT-PCR. Across the 6-kb region, only a polymorphic 49-bp indel and a linked single nucleotide polymorphism (SNP) in the *MtnA* 3’ UTR showed a large difference in frequency between the populations, being this deletion present in 10 of the 12 European lines, but absent in Africa (Fig 4A). This indel was previously observed to segregate in natural populations from North America [20]. A comparison with three outgroup species (*D*. *sechellia*, *D*. *simulans*, and *D*. *yakuba*) indicated that the deletion was the derived variant. The qRT-PCR data revealed that the two European lines that lacked the deletion had *MtnA* expression that was similar to that of the African lines, but much lower than the other European lines. This result held for both brain and gut expression. Taken together, these results suggest that the 3' UTR polymorphism contributes to *MtnA* expression variation in natural populations. Furthermore, the expression variation is not limited to the brain, but shows a correlated response in at least one other tissue (Fig 4B).

### Functional test of the effect of the *MtnA* 3' UTR polymorphism on gene expression

**Fig 5:**
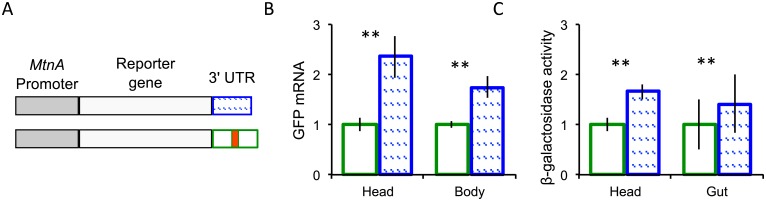
Reporter gene constructs and their expression. (A) The gray boxes represent the *MtnA* promoter, which is identical between the African and European alleles. The white boxes represent the GFP/*lacZ* reporter genes. The blue hatched box represents the *MtnA* 3’ UTR with the deletion. The green box represents the *MtnA 3’* UTR with the additional 49 bp marked in red. The same color scheme applies to the bar plots. (B) The two versions of the GFP reporter gene differ significantly in expression in heads (*t*-test, *P* = 0.0019) and bodies (*t*-test, *P* = 0.0046), as assayed by qRT-PCR. (C) The two versions of the *lacZ* reporter gene differed significantly in expression in heads (*t*-test, *P* = 0.0006) and guts (*t*-test, *P =* 0.0001) as measured by β-galactosidase enzymatic activity. The error bars represent the standard error of the mean.

To test if the 49-bp deletion in the *MtnA* 3' UTR has an effect on gene expression, we designed expression constructs in which the *MtnA* promoter was placed upstream of either a green fluorescent protein (GFP) or *lacZ* reporter gene. Two versions of each reporter gene were made, one with the ancestral *MtnA* 3' UTR sequence and one with the derived *MtnA* 3' UTR sequence, which has the 49-bp deletion (Fig 5A). The reporter genes were then introduced into the *D*. *melanogaster* genome by PhiC31 site-specific integration [22,23].

Our analysis of *MtnA* expression in the brain and gut indicated that the difference in expression observed between African and European populations is not brain-specific (Fig 4B). This is further supported by the expression of the reporter gene constructs. For the GFP reporter gene, the presence of the 3’ UTR deletion led to increased expression in both the brain and body (Fig 5B), with the difference in expression being 2.3-fold and 1.75-fold, respectively. A similar result was found for the *lacZ* reporter gene, where the 3’ UTR deletion led to 1.7-fold and 1.4-fold higher expression in the head and gut, respectively (Fig 5C).

### *MtnA* expression in the brain

**Fig 6:**
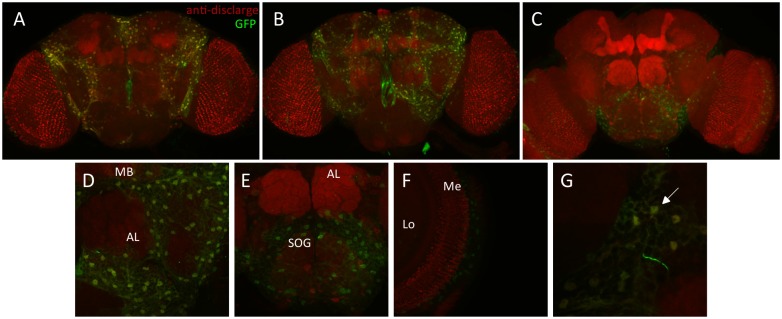
Expression of an *MtnA*-GFP reporter gene in the brain. (A-C) GFP expression driven by the reporter gene construct with the ancestral *MtnA* 3’ UTR variant. (D-G) Higher magnification of the brain regions where GFP is expressed. AL: antennal lobe, MB: mushroom bodies, SOG: subesophageal ganglion, Lo: lobula, Me: medulla. In (G) the arrow indicates cells expressing GFP. Green: GFP, red: anti-disclarge, targeting general neuropil.

*MtnA* shows high expression in most *D*. *melanogaster* organs, including the fat body, digestive system, Malpighian tubule, and brain [24]. Although it has been documented that *MtnA* and its paralogs are involved in heavy metal homeostasis and tolerance, it is poorly understood which other functions *MtnA* might have and in which cells it is expressed. To get a more detailed picture of *MtnA* expression in the brain, we examined the expression of the GFP reporter gene by confocal imaging of dissected brain tissue (Fig 6).

GFP expression driven by the *MtnA* promoter is evident in cells that form a mesh-like structure surrounding the brain and in between the neuropiles (Fig 6). *MtnA* does not appear to be expressed at a discernible level in neurons, as the cells expressing GFP do not have dendrites or axonal processes. The shape and localization of the cells expressing GFP in the brain suggest that they are glia, which provide neurons with developmental, structural and trophic support as well as with protection against toxic elements [25–27]. In a genome-wide expression profiling study it was found that *MtnA* is expressed in the astrocyte glial cells of larvae and adults of *D*. *melanogaster* [28]. Although we cannot be certain that *MtnA* expression is limited to the glia in the brain, our results provide direct evidence that *MtnA* is expressed in cell types other than the copper cells of the midgut and Malpighian tubules, as previously reported [29].

### Frequency of the *MtnA* 3' UTR deletion in additional populations

To better characterize the geographical distribution of the indel polymorphism in the *MtnA* 3' UTR, we used a PCR-based assay to screen ten additional *D*. *melanogaster* populations across a latitudinal range spanning from tropical sub-Saharan Africa to northern Europe (Table 1). We found that the deletion was at very low frequency in sub-Saharan Africa, but nearly fixed in populations from northern Europe. This suggests that, at least outside of the ancestral species range, there is a latitudinal cline in the deletion frequency. Indeed, when the sub-Saharan populations are excluded, there is a highly significant correlation between latitude and deletion frequency (linear regression; *R* = 0.95, *P* = 0.0004). This correlation still holds when the sub-Saharan populations are included (using the absolute value of latitude), but is weaker (*R* = 0.80, *P* = 0.001).

**Table 1:** Frequency of the *MtnA* 3’ UTR deletion in different populations.

Population	*N*	Latitude	Frequency of deletion [95% CI]
Sweden	12	63.8 N	1.00 [0.86–1.00]
Denmark	12	55.7 N	0.96 [0.80–1.00]
The Netherlands	12	52.2 N	0.83 [0.64–0.94]
Germany	11	48.1 N	0.91 [0.73–0.98]
France	12	45.8 N	0.92 [0.75–0.98]
Cyprus	10	35.1 N	0.65 [0.43–0.83]
Egypt	14	30.1 N	0.60 [0.42–0.77]
Cameroon	6	6.3 N	0.00 [0.00–0.26]
Malaysia	12	3.1 N	0.45 [0.27–0.65]
Rwanda	12	2.5 S	0.08 [0.02–0.25]
Zambia	10	16.5 S	0.05 [0.01–0.24]
Zimbabwe	11	17.3 S	0.00 [0.00–0.15]

*N*, number of lines. Because the deletion was polymorphic in some lines, its frequency was calculated on the basis of two alleles per line.

To investigate if the clinal distribution of the *MtnA* 3’ UTR deletion is present on other continents, we analyzed pooled sequencing (pool-seq) data from North America and Australia [30,31]. In North America, there is a significant correlation between latitude and deletion frequency (*R* = 0.94, *P* = 0.005) (Table 2). A similar pattern was seen in Australia, although data from only two populations were available. The deletion is at a frequency of 42% in Queensland (latitude 16 S) and 61% is Tasmania (latitude 42 S). The difference in deletion frequency between the two populations is significant (Fisher’s exact test, *P* = 0.02).

**Table 2:** Frequency of the *MtnA* 3’ UTR deletion in North American populations.

Population	*N*^a^	Latitude	Total reads^b^	*MtnA* 3’ UTR reads^c^	Deletion reads^d^	Frequency of deletion [95% CI]
Maine	322	45.5 N	125.8	301	171	0.57 [0.51–0.62]
Pennsylvania	900	40.0 N	593.9	1400	743	0.53 [0.50–0.56]
North Carolina	92	35.5 N	47.1	67	32	0.48 [0.36–0.60]
South Carolina	96	33.0 N	81.8	255	107	0.42 [0.36–0.48]
Georgia	102	30.9 N	96.9	246	101	0.41 [0.35–0.47]
Florida	174	25.5 N	103.7	225	76	0.34 [0.28–0.40]

^a^ Number of autosomes in the pooled sample (including all replicates)

^b^ Number of paired reads for the whole genome (in millions)

^c^ Number of reads that mapped to the *MtnA* 3’ UTR

^d^ Number of reads that matched the *MtnA* 3’ UTR deletion allele

### Evidence for positive selection at the *MtnA* locus

To test for a history of positive selection at the *MtnA* locus, we performed a population genetic analysis of the 6-kb *MtnA* region in the original European (the Netherlands) and African (Zimbabwe) population samples. In addition, we sequenced this region in 12 lines of a Swedish population, in which the 49-bp 3' UTR deletion was at a frequency of 100% (Table 1). Across the entire region, the Zimbabwean population showed the highest nucleotide diversity, having 1.43- and 2.50-fold higher values of *π* than the Dutch and Swedish populations, respectively (Table 3). Tajima’s *D* was negative in all three populations, and was significantly negative in both Zimbabwe and the Netherlands (Table 3). This could reflect a history of past positive or negative selection at this locus, but could also be caused by demographic factors, such as population expansion.

**Table 3:** Summary statistics for the *MtnA* locus.

Population	*n*	*S*	*θ*	*π*	*TajD*	*nHap*
Zimbabwe	11	54	0.312	0.194	-1.89*	11
The Netherlands	12	41	0.231	0.138	-1.85*	11
Sweden	12	17	0.096	0.078	-0.83	9

*n*, number of sequences; *S*, number of segregating sites; *θ*, Watterson’s estimate of nucleotide diversity (per 100 sites) [32]; *π*, mean pairwise nucleotide diversity (per 100 sites) [33]; *TajD*, Tajima’s *D* [34]; *nHap*, number of haplotypes.

*P < 0.05.

**Fig 7:**
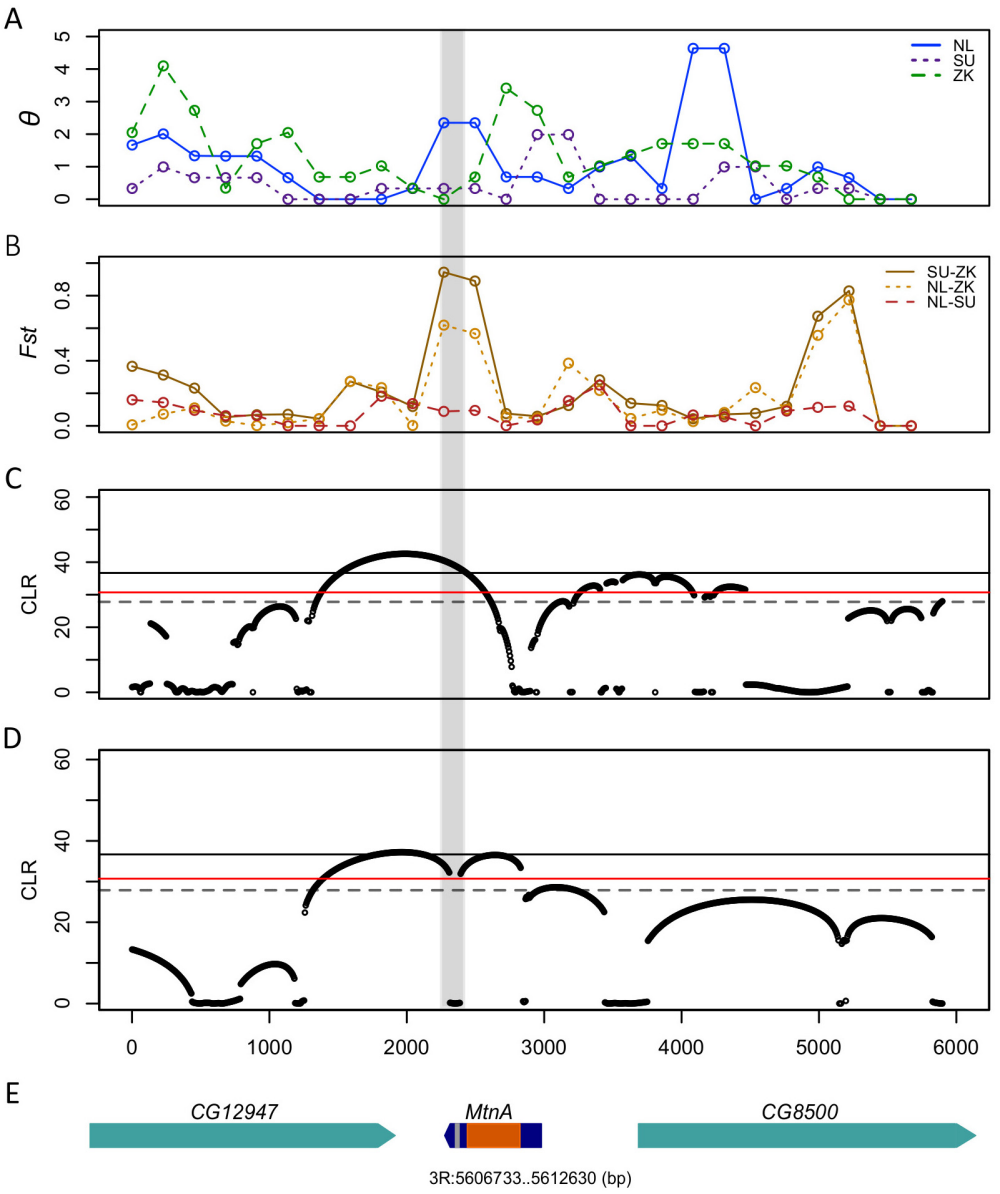
Evidence for positive selection at the *MtnA* locus. (A) Watterson’s *θ* of *D*. *melanogaster* populations from Zimbabwe (ZK), the Netherlands (NL) and Sweden (SU) calculated in sliding windows of 500 bp with a step size of 250 bp. (B) *F*_st_ values for pairwise comparisons of ZK, NL and SU calculated in sliding windows of 500 bp with a step size of 250 bp. (C) Selective sweep *(SweepFinder)* analysis of the Netherlands population showing the composite likelihood ratio (CLR) statistic in sliding windows of 1000 bp. (D) Selective sweep (*SweepFinder*) analysis of the Swedish population showing the CLR statistic in sliding windows of 1000 bp. The black line indicates the 5% significance threshold calculated using the demographic model of Duchen et al. [5] for neutral simulations. The red line indicates the 5% significance threshold calculated using the demographic model of Werzner et al. [6] for neutral simulations and the gray dashed line indicates the 5% significance threshold using the model of Thornton and Andolfatto [35]. (E) Gene models for the 6-kb region analyzed. The gray highlighted region indicates the position of the 49-bp indel polymorphism in the *MtnA* 3’ UTR.

A sliding window analysis was performed to determine the distribution of nucleotide diversity (*θ*) (Fig 7A) and population differentiation (*F*_st_) (Fig 7B) across the *MtnA* region. The region flanking the 3’ UTR indel polymorphism showed very low sequence variation in Zimbabwe and Sweden, but higher variation in the Netherlands. This pattern is due to the fact that the ancestral state of the indel polymorphism is fixed in the Zimbabwean population and the derived state is fixed in the Swedish population. In the Dutch population, the *MtnA* 3’ UTR is polymorphic for the deletion (two of the 12 lines have the ancestral state). This leads to higher nucleotide diversity than in the Swedish population, because the ancestral, non-deletion alleles contain more SNPs than the derived, deletion alleles. On average, Sweden and Zimbabwe showed the greatest population differentiation, with *F*_st_ reaching a peak in the 3’ UTR of *MtnA*, whereas values of *F*_st_ were lowest for the comparison of the Dutch and Swedish populations, indicating that there is very little differentiation between them (Fig 7b).

If positive selection has favored the derived *MtnA* allele (with the 49-bp 3' UTR deletion) in northern populations, then in this region of the genome one would expect there to be less variation among chromosomes containing the deletion than among those with the ancestral form of the allele. Indeed, this is what we observe in the Netherlands, where both alleles are segregating. Across the 6-kb region, there are 41 segregating sites within the Dutch population (Table 3). Among the 10 chromosomes with the deletion, there are 18 segregating sites, while between the two chromosomes lacking the deletion there are 23 segregating sites. This indicates that chromosomes with the deletion, which are in high frequency, shared a much more recent common ancestor. To test if this pattern differs from that expected under neutral evolution, we performed the Hudson's haplotype test (HHT) [36] using three different demographic models of the *D*. *melanogaster* out-of-Africa bottleneck for neutral simulations. Under the model of Werzner et al. [6], HHT was significant (*P* = 0.031). Under the models of Thornton and Andolfatto [35] and Duchen et al. [5], HHT was marginally significant (*P* = 0.076 and *P* = 0.094, respectively). These results suggest that neutral evolution and demography are unlikely to explain the observed patterns of DNA sequence variation.

To further test if the *MtnA* locus has experienced recent positive selection in northern Europe, we used the composite likelihood ratio (CLR) test to calculate the likelihood of a selective sweep at a given position in the genome, taking into account the recombination rate, the effective population size, and the selection coefficient of the selected mutation [37,38]. Within the Dutch population, the CLR statistic shows a peak in the region just adjacent to the *MtnA* 3' UTR deletion (Fig 7C). This peak was significant when the demographic models of Duchen et al. [5], Werzner et al. [6], and Thornton and Adolfatto [35] were used for neutral simulations, which provides compelling evidence for a recent selective sweep at the *MtnA* locus in the Netherlands population. A similar result was obtained for the Swedish population (Fig 7D), where the CLR statistic was above the 5% significance threshold determined from all three of the bottleneck models, suggesting that the selective sweep was not limited to a single population, but instead affected multiple European populations.

To test the possibility that the deletion in the *MtnA* 3’ UTR might have risen to high frequency as a result of hitchhiking with another linked polymorphism, we examined linkage disequilibrium (LD) across a 100 kb region flanking the *MtnA* locus in the Netherlands population (S1 Fig). The degree of linkage disequilibrium, *r*^2^ [39], was calculated between all pairs of SNPs present in the 100 kb region, excluding singletons. The SNP corresponding to the indel polymorphism (Fig 4a), position 53 of the linkage disequilibrium matrix, is not in significant LD with any of the 94 SNPs present along the 100 kb region analyzed (S1 Fig). These results indicate that the high frequency of the *MtnA* 3’ UTR deletion cannot be explained by linkage with another positively selected locus.

### Association of the *MtnA* 3' UTR deletion with increased oxidative stress tolerance

**Fig 8:**
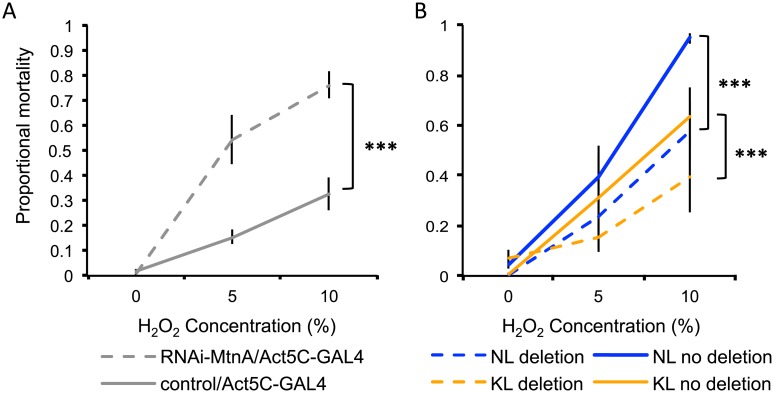
Proportional mortality after oxidative stress. (A) RNAi-mediated *MtnA* knockdown (hatched lines) and control flies (solid lines). *P*-values are shown for within population/background comparisons. (B) Dutch (blue) and Malaysian (orange) flies with the deletion (hatched lines) and without the deletion (solid lines) in the *MtnA* 3’ UTR. Error bars indicate the standard error of the mean. *P < 0.05, **P < 0.01, ***P < 0.005.

**Fig 9:**
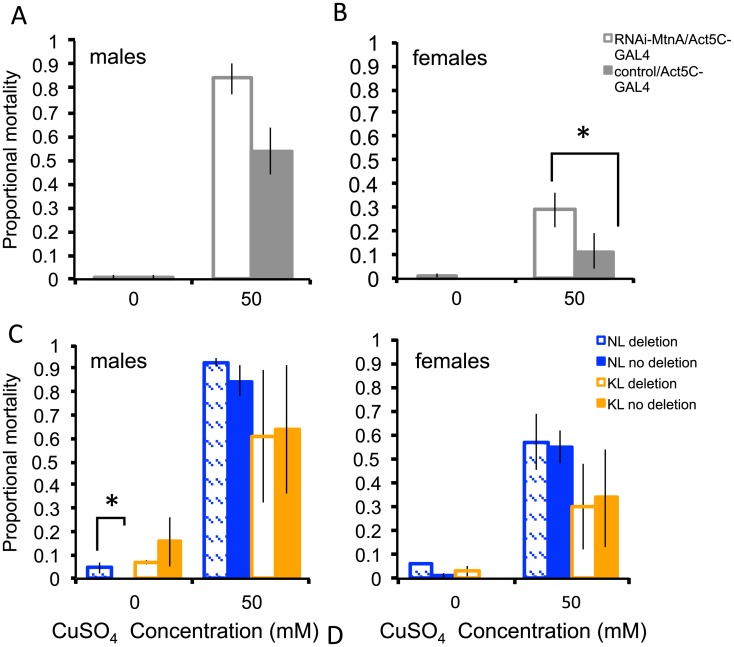
Proportional mortality after copper sulphate exposure. (A,B) Copper tolerance in RNAi-mediated *MtnA* knockdown flies (white, RNAi-*MtnA*/Act5C-GAL4) and control flies expressing normal levels of *MtnA* (solid grey, control/Act5C-GAL4). (C) Male and (D) female flies from the Dutch (NL, blue) and the Malaysian (KL, orange) population with the deletion (hatched) and without the deletion (solid). *P*-values are shown for within population/background comparisons. Error bars indicate the standard error of the mean. *P < 0.05, **P < 0.01, ***P < 0.005.

*MtnA* expression has been linked to increased heavy metal tolerance [19,20,40] and metallothioneins in general have been associated with protection against oxidative stress [18,41]. To test if *MtnA* plays a role in oxidative stress and/or heavy metal tolerance, we used RNA interference (RNAi) to knockdown *MtnA* expression; these flies, along with their respective controls, were exposed to either hydrogen peroxide or copper sulfate. A knockdown in *MtnA* expression was significantly associated with increased mortality in the presence of hydrogen peroxide (*P* < 0.001; Fig 8A) and copper sulphate (*P* = 0.026; Fig 9A and 9B), although for the latter, this decrease was only significant in females.

To further test if the deletion in the *MtnA* 3’ UTR could be associated with an increase in oxidative stress and/or heavy metal tolerance, a subset of *D*. *melanogaster* lines from the Dutch and Malaysian populations, either with or without the deletion, were exposed to hydrogen peroxide and copper sulfate. The 3’ UTR deletion was associated with a significant increase in survival in the presence of hydrogen peroxide in both the Dutch (*P* = 0.001; Fig 8B) and Malaysian (*P* = 0.001; Fig 8B) populations. The 3’ UTR deletion had no significant effect on survival in the presence of copper sulfate in Dutch and Malaysian females (*P* = 0.976 and *P* = 0.732 respectively; Fig 9D) or males (*P* = 0.578 and *P* = 0.904 respectively; Fig 9C). Thus, the deletion in the *MtnA* 3’ UTR was associated with increased oxidative stress tolerance, but not increased heavy metal tolerance.

## Discussion

Differential expression of *MtnA* between a European and an African population of *D*. *melanogaster* was first detected in a brain-specific RNA-seq analysis [12]. In the present study, we confirm this inter-population expression difference by qRT-PCR and show that it is associated with an indel polymorphism in the *MtnA* 3’ UTR. We also perform reporter gene experiments to demonstrate that a large proportion of the expression difference can be attributed to this indel polymorphism. The ancestral state of the 3’ UTR contains a 49-bp sequence that is deleted in a derived allele that is present in worldwide populations. The deletion is nearly absent from sub-Saharan Africa, but present in frequencies >80% in northern Europe (Table 1). The deletion is present at intermediate frequency in Egypt (60%), Cyprus (65%) and Malaysia (45%). These findings suggest that positive selection has favored the 3' UTR deletion, at least within northern European populations. This interpretation is supported by population genetic analyses that indicate a recent selective sweep at the *MtnA* locus in populations from the Netherlands and Sweden (Fig 7). Furthermore, a clinal relationship between deletion frequency and latitude is also seen in North America and Australia, suggesting that there is a common selection gradient affecting all populations outside of sub-Saharan Africa.

Although chromosome arm 3R is known to harbor inversion polymorphisms that vary in frequency with latitude in cosmopolitan populations [42], we can rule out linkage to a segregating inversion as a cause for the clinal pattern seen for the *MtnA* 3’ UTR deletion. A previous analysis of the same Dutch population used in our study found that only one of the isofemale lines harbored an inversion on 3R, *In(3R)P* [43]. This was line *NL13*, which is one of the 10 lines with the *MtnA* 3’ UTR deletion (Fig 4A). Thus, there is no evidence for linkage between the inversion and the deletion. Moreover, the *MtnA* gene lies 7 Mb outside of the nearest breakpoint of *In(3R)P*.

Using hydrogen peroxide tolerance assays, we found evidence that knocking down *MtnA* expression decreases oxidative stress tolerance (Fig 8B). The association of the deletion in the *MtnA* 3’ UTR with increased survival in the presence of hydrogen peroxide (Fig 8A) suggests that the deletion has been selectively favored in some environments because it confers increased tolerance to oxidative stress. While cytotoxic reactive oxygen species (ROSs) are generated by natural metabolic processes, they can also be introduced via abiotic factors in the environment, such as radiation, UV light or exposure to toxins. The significant correlation between the frequency of the 3' UTR deletion and latitude, coupled with its association with increased oxidative stress tolerance suggests that environmentally induced oxidative stress may vary clinally, with greater stress in northern European environments. Regulation of the oxidative stress response usually occurs via upregulation of antioxidant protective enzymes in response to the binding of a *cis-*acting *antioxidant-responsive element* (ARE), which contains a characteristic sequence to which stress-activated transcription factors can bind [41]. A recent example of adaptation to oxidative stress in *Drosophila* is the insertion of the *Bari-Jheh* transposable element into the intergenic region of *Juvenile Hormone Epoxy Hydrolase* (*Jheh*) genes, which adds additional AREs that upregulate two downstream *Jheh* genes and was associated with increased oxidative stress tolerance [44]. Interestingly, the *Bari-Jheh* insertion also shows evidence for a partial selective sweep in non-African *D*. *melanogaster* [45], suggesting that oxidative stress may have imposed an important selective constraint on the colonization of Europe. However, the *MtnA* 3’ UTR deletion cannot mediate its associated increase in oxidative stress tolerance in a similar way, since it does not add any new AREs.

Due to their high inducibility in response to heavy metals, metallothioneins have traditionally been thought to play a role as detoxifiers specifically of heavy metals. However, this view has come into question recently, and metallothioneins are now thought to be a part of the general stress response and may function as scavengers of free radicals [41]. The association of the *MtnA* 3’UTR deletion with increased oxidative stress tolerance (Fig 8A) is in line with this more recent view of the role of metallothioneins, while the observed increased mortality after copper exposure in females in which *MtnA* expression has been knocked down (Fig 9D) is in keeping with the more traditional view. However, we found no association between the presence of the deletion and copper tolerance. This may be because the RNAi knockdown results in an *MtnA* expression level that is much lower than that of naturally occurring alleles, and copper tolerance is only affected when *MtnA* expression falls below a minimal threshold. The precise mechanisms of how metallothioneins interact with other metal processing systems after their initial binding and help remove excess of heavy metals, remain unclear [41].

At present, the mechanism by which the 3' UTR deletion affects *MtnA* gene expression is unknown. Although the deletion appears to have an effect on the usage of the *MtnA-RB* transcript isoform (S1 Table), this isoform is too rare (<2% of all *MtnA* transcripts) to account for the observed 4-fold difference in *MtnA* expression. Another possibility is that the deleted 3' UTR region contains one or more binding sites for a microRNA (miRNA). miRNAs are short, non-coding RNAs that modulate the expression of genes by inhibiting transcription or inducing mRNA degradation [46]. They are known to bind to a seed region that consists of 6–8 nucleotides in the 3’ UTR of their target mRNA. Post-transcriptional gene expression regulation by miRNAs can result in the fine-tuned regulation of a specific transcript or can cause the complete silencing of a gene in a particular tissue or developmental stage [46–48]. To identify miRNAs that might bind specifically to the 49-bp sequence present in the ancestral form of the *MtnA* 3’ UTR, we used the UTR predictor [49]. The UTR predictor takes into account the three-dimensional structure of the miRNA and the 3’ UTR, as well as the energetic stability of the miRNA-3’ UTR base-pair binding. The score given by the UTR predictor is an energetic score, with the most negative scores indicating the most probable interactions. Our analysis of the *MtnA* 3' UTR identified five candidate miRNAs with scores below -6 that had predicted binding sites overlapping with the 49-bp indel region (Table 4). These candidates should serve as a good starting point for future functional tests of putative miRNA-3' UTR interactions.

**Table 4:** Top scoring miRNAs predicted to bind within the polymorphic 49-bp sequence in the *MtnA* 3' UTR.

microRNA	Binding position	binding sites	Seed	ddG
*dme-miR-284*	52	1	8:0:1	-12.68
*dme-miR-954*	102	1	8:1:0	-10.61
*dme-miR-956*	43	1	8:1:1	-6.39
*dme-miR-9c*	74	1	8:1:1	-6.13
*bantam*	52	1	8:1:1	-6.13

The binding position coordinate indicates the distance in base pairs between the start of the 3' UTR and first miRNA binding site. The notation describing the seed (X:Y:Z) represents the size of the seed (X), the number of mismatches (Y) and the number of G:U wobble pairs (Z). The energetic score for the probability and stability of the binding is denoted by ddG. The more negative the score is, the more probable is the interaction between the 3’ UTR and the miRNA.

Genetic variation provides the substrate upon which natural selection acts, resulting in an increase in the frequency of alleles that are beneficial in a given environment. Because changes in gene expression, especially those caused by variation in *cis*-regulatory elements, are predicted to have fewer pleiotropic effects than changes occurring within coding regions, it has been proposed that they are the most frequent targets of positive selection [50–52]. In contrast to structural changes in protein sequences, changes in gene expression can be specific to a particular a tissue or developmental stage. Our results indicate that the observed variation in *MtnA* expression is not specific to the brain, as a similar expression pattern is also seen in the gut (Fig 4). This suggests that the 3' UTR deletion has a general effect on *MtnA* expression, which is present at high levels in almost all organs of *D*. *melanogaster* [24]. However, tissue-specific effects of the difference in *MtnA* expression cannot be ruled out. As shown in Fig 6, GFP expression driven by the *MtnA* promoter in the brain is limited to what seems only one cell type, which according to their morphological and anatomical characteristics, could correspond to glia. It has been reported that glia cells protect neurons and other brain cells from ROS damage caused by oxidative stress [53,54] and the fact that *MtnA* has been found to be expressed in the astrocyte glial cells in larva and adult flies [28], suggests that *MtnA* expression in glia could serve as neuronal protection against environmental factors, such as exposure to xenobiotics, that trigger an oxidative stress response [29,55–57]. Our functional experiments showing an association between genetic variation in *MtnA* and oxidative stress tolerance are consistent with *MtnA* expression in glia providing protection against oxidative stress, which may be especially important in the brain, as neurons are highly susceptible to ROS damage.

## Materials and Methods

### Fly strains

This study used isofemale lines from 12 populations of *D*. *melanogaster*, including: Zimbabwe (Lake Kariba), Zambia (Lake Kariba), Rwanda (Gikongoro), Cameroon (Oku), Egypt (Cairo), Cyprus (Nicosia), Malaysia (Kuala Lumpur), France (Lyon), Germany (Munich), the Netherlands (Leiden), Denmark (Aarhus) and Sweden (Umeå). The lines from Zimbabwe and the Netherlands were the same as those used in previous expression studies [10–12]. Flies from Germany were collected from different locations in the greater Munich area. Flies from Cyprus were collected from a single location near Nicosia. Flies from Denmark were kindly provided by Volker Loeschcke (Aarhus University). Flies from Sweden and Malaysia were kindly provided by Ricardo Wilches and Wolfgang Stephan (University of Munich). The remaining fly lines were collected as part of the *Drosophila* Population Genomics Project [8] and were kindly provided by John Pool and Charles Langley (University of California, Davis).

Flies expressing hairpin RNA targeted against *MtnA* mRNA under the control of the GAL4/UAS system (RNAi-*MtnA*; transformant ID: 105011) and the host line used in their creation (control; transformant ID: 60100) were obtained from the Vienna *Drosophila* Stock Center [58] *Act5C*/*Cyo* flies expressing GAL4 under the control of an *Act5C* driver were kindly provided by Ilona Grunwald Kadow. For tolerance assays, *Act5C*/*Cyo* females were crossed to RNAi-*MtnA* and control males and the progeny (RNAi-*MtnA*/*Act5C*-GAL4 and control/*Act5C*-GAL4) were used in tolerance assays. Using qRT-PCR as described below, *MtnA* expression was confirmed to be knocked down by 90.03% in males and 87.58% in females in RNAi-*MtnA*/*Act5C*-GAL4 flies in comparison to control/*Act5C*-GAL4. Flies were maintained on standard cornmeal-molasses medium at a constant temperature of 22° with a 14 hour light/10 hour dark cycle.

### Quantitative reverse transcription PCR (qRT-PCR)

Validation of the *MtnA* expression results obtained from brain RNA-seq data [12] was performed by qRT-PCR using TaqMan probes (Applied Biosystems, Foster City, California, USA). For population-level comparisons, six brains were dissected from males and females of each of the 11 lines from Zimbabwe (*ZK84*, *ZK95*, *ZK131*, *ZK145*, *ZK157*, *ZK186*, *ZK191*, *ZK229*, *ZK377*, *ZK384*, *ZK398*) and five brains were dissected from males and females of each of the 12 lines from the Netherlands (*NL01*, *NL02*, *NL11*, *NL12*, *NL13*, *NL14*, *NL15*, *NL16*, *NL17*, *NL18*, *NL19*, *NL20*). The dissected brains of each population and sex were pooled following the RNA-seq strategy previously described [12]. The above procedure was repeated in two biological replicates for each sex and population. To compare the *MtnA* expression of individual lines within populations, subsets of eight lines were chosen from Zimbabwe (*ZK84*, *ZK95*, *ZK131*, *ZK145*, *ZK157*, *ZK186*, *ZK377*, *ZK384*) and the Netherlands (*NL01*, *NL02*, *NL11*, *NL12*, *NL15*, *NL16*, *NL17*, *NL18*). Thirty whole brains and digestive tracts (from foregut to hindgut) were dissected per line. Two biological replicates of each line (each consisting of 30 brains or guts) were processed. Tissue was dissected from flies 4–6 days old in 1X PBS (phosphate buffered saline). The tissue was stored in RNAlater (Life Technologies, Carlsbad, CA, USA) at -80° until RNA extraction. Total RNA extraction and DNase I digestion was performed using the MasterPure RNA Purification Kit (Epicentre, Madison, WI, USA). One microgram of total RNA was reverse transcribed using random primers and SuperScript II reverse transcriptase (Life Technologies) following the manufacturer’s instructions. TaqMan gene expression assays (Applied Biosystems) were used for *MtnA* (Dm02362764_s1) and *RpL32* (Dm02151827_g1). qRT-PCR was performed using a Real-Time thermal cycler CFX96 (Bio-Rad, Hercules, CA, USA). Two biological replicates, each with two technical replicates, were processed for each sample. The ΔΔCt method was used to compute the normalized expression of *MtnA* using the ribosomal protein gene *RpL32* as the reference [59].

### CNV assays

The paralogous genes *AttacinA* (*AttA*) and *AttacinB* (*AttB*) were used as positive controls for CNV assays, because they share 97% nucleotide identity [21] and can be co-amplified with the same primer set. The sequences for *AttA* and *AttB* were downloaded from FlyBase [60] and aligned using the ClustalW2 algorithm implemented in SeaView (version 4) [61]. Primers were designed for the second coding exon, where the nucleotide identity of *AttA* and *AttB* is 100%. The primer sequences were as follows: forward (5’-GGTGCCTCTTTGACCAAAAC-3’) and reverse (5’-CCAGATTGTGTCTGCCATTG-3’). The ribosomal protein gene *RpL32*, which is not known to show CNV, was used as a negative control. The *RpL32*-specific primers were: forward (5’-GACAATCTCCTTGCGCTTCT-3’) and reverse (5’-AGCTGGAGGTCCTGCTCAT-3’). The primers specific for *MtnA* were: forward (5’-CACTTGACCATCCCATTTCC-3’) and reverse (5’-GGTCTGCGGCATTCTAGGT-3’). CNV was assessed among 12 lines from the Netherlands and 11 lines from Zimbabwe. Individual DNA extractions were performed separately for three flies of each line and copy number was assessed individually for each fly. Genomic DNA was extracted using the MasterPure DNA Purification Kit (Epicentre). The assessment of CNV from genomic DNA was done with iQ SYBR Green Supermix (Bio-Rad) following the manufacturer’s instructions. CNV assays were performed using a Real-Time thermal cycler CFX96 (Bio-Rad). The relative copy numbers of *MtnA* and *AttA*/*AttB* were obtained by the ΔCt method using *RpL32* as the reference gene.

### Sequencing of the *MtnA* locus

Approximately 6 kb of the *MtnA* genomic region, spanning from the second intron of *CG12947* to the 3’ UTR of *CG8500* (genome coordinates 3R: 5,606,733–5,612,630), were sequenced in 12 Dutch, 11 Zimbabwean and 12 Swedish lines (Fig 1). The following primer pairs were used (all 5’ to 3’): GATGGTGGAATACCCTTTGC and AAAGCGGGTTTACCAGTGTG; GTTGGCCTGGCTTAATAACG and ACTGGCACTGGAGCTGTTTC; GCTCTTGCTAGCCATTCTGG and AGAACCCGGCATATAAACGA; GATATGCCCACACCCATACC and GTAGAGGCGCTGCATCTTGT; CACTTGACCATCCCATTTCC and CAAGTCCCCAAAGTGGAGAA; CTTGATTTTGCTGCTGACCA and ATCGCCACGATTATGATTGC; CAGGACAATCAAGCGGAAGT and TTATGAAGCGCAGCACCAGT; GACCCACTCGAATCCGTATC and TGCTTCTTGGTGTCCAGTTG. PCR products were purified with ExoSAP-IT (Affymetrix, Santa Clara, CA, USA) and sequenced using BigDye chemistry on a 3730 automated sequencer (Applied Biosystems). Trace files were edited using Sequencher 4.9 (Gene Codes Corporation, Ann Arbor, MI, USA) and a multiple sequence alignment was generated using the ClustalW2 algorithm in SeaView (version 4) [61]. All sequences have been submitted to GenBank/EMBL under the accession numbers KT008059–KT008093.

### *MtnA* indel polymorphism screening and latitude correlation study

For individual flies of the isofemale lines described above, the presence or absence of the *MtnA* 3' UTR deletion was assessed by performing a two-step PCR (35 cycles of 98° for 5 sec. and 60° for 10 sec.) using the following primers: forward (5’-GCCGCAGACCAATTGATTA-3’) and reverse (5’-TTCTTTCCAGGATGCAAATG-3’). The frequency of the deletion was estimated on an allelic basis, as heterozygous individuals were detected in some populations. Binomial 95% confidence intervals were calculated for the frequency of the deletion using the probit method implemented in R [62]. The strength and significance of the correlation between the frequency of the deletion and latitude was determined using linear regression.

To determine the frequency of the *MtnA* 3’ UTR deletion on other continents, raw pool-seq reads from North America [30] and Australia [31] were downloaded from the National Center for Biotechnology Information (NCBI) short read archive (SRA). The reads were mapped to either the ancestral or derived (with 49-bp deletion) version of the *MtnA* 3’ UTR using NextGenMap [63]. Only reads spanning the site of the indel were considered informative. The deletion frequency was estimated as the proportion of informative reads that matched the deletion allele. The 95% confidence interval was estimated using the probit method in R [55].

### Cloning and transgenesis

To test whether the indel polymorphism found in the *MtnA* 3’ UTR can account for the difference in expression observed between the Dutch and the Zimbabwean populations, we constructed transgenic flies using the phiC31 transgenesis system [23]. Two expression vectors containing a green fluorescent protein (GFP) reporter gene were constructed. *MtnA* 3’ UTR sequences from the Netherlands (line *NL20*) and Zimbabwe (line *ZK84*), corresponding to chromosome arm 3R coordinates 5,607,448–5,611,691, were PCR-amplified with forward (5’-TTTCCTCGAACTTGTTCACTTG -3’) and reverse (5’- GCCCGATGTGACTAGCTCTT -3’) primers and cloned into the p*CR2*.*1-TOPO* vector (Invitrogen). The promoter region of *MtnA* (corresponding to genome coordinates 3R: 5,607,983–5,612,438), which is identical in the Dutch and the Zimbabwean populations, was also PCR amplified and cloned separately into the p*CR2*.*1-TOPO* vector using forward (5’-GCCGCAGACCAATTGATTA-3’) and reverse (5’-TTCTTTCCAGGATGCAAATG-3’) primers. To generate the GFP expression construct, the *MtnA* promoter was excised with *EcoR*I and ligated into the *EcoR*I site at the 5’ end of GFP in the plasmid p*RSET/EmGPP* (Invitrogen). Using *Ava*I and *Xba*I, the fragment containing the *MtnA* promoter and GFP was excised from the p*RSET/EmGPP* plasmid and ligated into the *Ava*I–*Xba*I sites proximal to the *MtnA* 3’ UTR in the p*CR2*.*1-TOPO* vector. The whole construct (promoter + GFP + 3’ UTR) was then excised with *Xba*I and *Kpn*I and ligated into the *Xba*I–*Kpn*I sites of the *pattB* integration vector [23]. For the *lacZ* constructs, the *MtnA* promoter was excised from the p*CR2*.*1-TOPO* vector with *EcoR*I and ligated into the *EcoR*I site 5’ of the *lacZ* coding sequence in the p*CMV-SPORT-βgal* plasmid (Life Technologies). PCR primers with overhangs containing restriction sites for *Xho*I and *Xba*I (forward 5’- GGTCCGACTCGAGGCGAAATACGGGCAGACATG -3’ and reverse 5’- GGTGCTCTAGAGCTCCATAGAAGACACCGGGAC -3’) were used to amplify the *MtnA* promoter/*lacZ* fragment and the product was ligated into the *Xho*I–*Xba*I sites just upstream of the *MtnA* 3’ UTR fragment in the p*CR2*.*1-TOPO* vector. Finally, the whole construct was excised using *Xba*I and *Kpn*I and ligated into the *Xba*I–*Kpn*I sites of the *pattB* vector (Fig 5). PhiC31 site-specific transgenesis was used to generate flies that differed only in the presence or the absence of the 49–bp sequence in the 3’ UTR of the reporter gene. The *M{vas-int*.*Dm}ZH-2A*, *M{3xP3-RFP*.*attP}ZH-51D* line was used for embryo microinjections. Microinjection and screening for transformants were carried out by Fly Facility (Clermont-Ferrand Cedex, France) and Rainbow Transgenic Flies (Camarillo, CA, USA). The successfully transformed flies were crossed to a *yellow*, *white* (*yw)* strain for two generations to eliminate the integrase.

### Reporter gene assays

#### GFP assays

The expression of the reporter gene GFP was measured in heterozygous flies generated by crossing transformant males to *yw* females. We tested for differences in the expression of GFP in bodies and heads separately. Differences in GFP expression between lines were tested by qRT-PCR. For this, total RNA was extracted from five bodies and ten heads of each transformant line using the RNA extraction and reverse transcription protocols described above. Thirteen biological replicates (six male and seven female) were processed for each line, each with two technical replicates. qRT-PCR was performed as described above using a custom Taqman probe for GFP (Applied Biosystems; forward primer: 5’-GAGCGCACCATCTTCTTCAAG-3’, reverse primer: 5’-TGTCGCCCTCGAACTTCAC-3’, FAM-labeled primer: 5’-ACGACGGCAACTACA-3’) and a probe for *RpL32* (Dm02151827_g1), which was used as an endogenous control. The data analysis was also performed as described above for *MtnA* gene expression. A *t*-test was performed to assess significance.

#### β-galactosidase assays

β-galactosidase activity was measured in groups of 30 heads or eight guts of homozygous transformant flies. Proteins were extracted from the tissues and the β-galactosidase activity assay was performed as described in [64] with the following exceptions. After dissection in cold PBS, tissues were frozen with liquid nitrogen and homogenized before the addition of 150 μL of the 0.1 M Tris-HCl, 1 mM EDTA, and 7 mM 2-mercaptoethanol buffer (pH 7.5). For each assay, two technical replicates of 60 μL of the supernatant containing the soluble proteins were combined with 50 μl of the 200 mM sodium phosphate (pH 7.3), 2 mM MgCl_2_, 100 mM 2-mecaptoethanol assay buffer. β-galactosidase activity was measured spectrophotometrically by following the change in absorbance at 420 nm at 37° Celsius. Four to five biological replicates were performed per tissue and per sex. Significance was assessed using a *t*-test.

### Brain confocal imaging

Brain tissue was dissected in ice-cold 1X PBS and fixed with PLP (8% paraformaldehyde in NaOH and PBS with lysine (1)-HCl) for one hour at room temperature as described in [65]. After fixation, the tissue was washed twice for 15 minutes with PBS-0.5% Triton X and then incubated for one hour in blocking solution (20% donkey serum, 0.5% Triton X in PBS) at room temperature. The primary antibody, mouse anti-disclarge (Developmental Studies Hybridoma Bank, University of Iowa, USA) was used at a 1:200 dilution and incubated overnight at 4° Celsius in blocking solution. After washing twice with PBS-0.5% Triton X, the tissue was incubated with the secondary antibody, 1:200 anti-rat-CY3 (Dianova, Hamburg, Germany). The brains were mounted in Vectashield mounting medium (Vector Laboratories, Burlingame, CA, USA) and scanned using confocal microscopy with a Leica SP5-2. The images were analyzed using the StackGroom plugin in ImageJ [66].

### Population genetic analysis and tests for selection

Summary statistics, including the number of segregating sites (*S*), number of haplotypes and Tajima’s *D* [34] were calculated using DnaSP v.5.10.1 [67]. The mean pairwise nucleotide diversity (*π*) [33], Watterson’s [32] estimate of nucleotide diversity (θ) and *F*_s*t*_ [68] were calculated as described in [5]. Hudson’s haplotype test (HHT) was carried out using *ms* [69] to perform coalescent simulations and *psubs* [70] to calculate the probability of observing a subset of *n* sequences containing *p* or fewer polymorphic sites. The demographic models of Thornton and Andolfatto [35], Duchen et al. [5], and Werzner et al. [6] were used to simulate the out-of Africa bottleneck.

To test for a selective sweep, a *SweepFinder* analysis was performed using the *SweeD* software [38]. The background site frequency spectrum (SFS) was calculated for the entire 3R chromosome arm using 11 whole genome sequences from the Netherlands population and one whole genome sequence from the French (Lyon) population [8]. The French sequence was included in order to have a constant sample size of 12 sequences for the calculation of the SFS. This approach did not bias the background, as the French sequence did not differ more from the Netherlands sequences than the Netherlands sequences did from each other (S2 Table, S2a Fig). Furthermore, the inclusion of a French line did not lead to a skew in the background SFS (S2b Fig). For the Swedish population, the background SFS of chromosome arm 3R was determined from 12 whole genome sequences from the Umeå population (S3 Table). In order to increase the power of the test, the invariant sites in the alignment were also included [37]. To assess the significance of the composite likelihood ratio (CLR) statistic, neutral simulations were performed using *ms* [69]. In the neutral simulations three demographic models were taken into account [5,6,35]. These models differ in several parameters, including: the timing of the out-of-Africa bottleneck, the current effective population sizes of the European and African populations, and the ancient demographic history of the African population. For our analyses, it is the estimated time of the out-of-Africa bottleneck that has the largest impact on the results. Duchen et al. [5] infer this bottleneck to have occurred around 19,000 years ago, Thornton and Andolfatto [35] around 16,000 years ago, and Werzner et al. [6] around 13,000 years ago. However, the 95% confidence intervals of the estimates are very wide, ranging from 7,359–43,000 years ago. Thus, the three estimates are not incompatible with each other. The recombination rate of the *MtnA* genomic region was obtained from the *D*. *melanogaster* recombination rate calculator [71]. A total of 10,000 simulations were performed. For each simulation, the maximum value of the CLR statistic was extracted and used to determine the 5% significance threshold. Linkage disequilibrium was calculated between all pairs of SNPs present using Lewontin’s *r*^*2*^ *= D*^*2*^*/p*_*1*_*q*_*1*_*p*_*2*_*q*_*2*_, where *D* is the frequency of the haplotypes and *p* and *q* represent the allele frequencies [39]. A fragment of ~100 kb flanking the *MtnA* locus (3R: 9,732,746..9,835,406) was analyzed, with singletons excluded. A Fisher’s exact test was used to assess significance of the *r*^2^ values.

### Copper and oxidative stress tolerance assays

Copper sulfate and hydrogen peroxide tolerance assays were performed using five *D*. *melanogaster* lines containing the *MtnA* 3’ UTR deletion (two Dutch and three Malaysian lines) and three lines without the deletion (two Malaysian and one Dutch line), as well as an *MtnA* knockdown line (RNAi-*MtnA*/*Act5C*-GAL4 and its control (control/*Act5C*-GAL4). Assays were performed at 25°C in tolerance chambers consisting of a plastic vial (diameter = 25 mm, height = 95 mm) with compressed cotton at the bottom containing 2.5 ml copper sulfate (Sigma Aldrich) or hydrogen peroxide (Sigma Aldrich) solution supplemented with 5% sucrose and sealed with a cork. Four to six day-old flies were separated by sex and tested in groups of 20. For each assay, one concentration of copper sulfate (50 mM) or two concentrations of hydrogen peroxide (5 or 10%) were tested with 5–7 replicates per sex and concentration. A control solution containing only sucrose was also tested with 3–5 (10–15 for *Act5C*-GAL4 background) replicates per sex for each assay. Mortality was recorded as the number of dead flies after 48 ± 1 hours. To determine the effect of the deletion, lines with and without the deletion were compared within each population or background. For copper sulfate assay analysis, *t*-tests were performed to assess significance. In order to account for potential differences in mortality inherent among the lines, proportional mortality data was scaled by mortality at 0 mM using the formula mortality/(1 + mean mortality at 0mM). For hydrogen peroxide assay analysis, the data for each assay and population was fit to a generalized linear model (GLM) using concentration, line, sex, and presence of the deletion as factors and a quasibinomial distribution using the glm function in R [62]. The tolerance results for each sex (S3 Fig) and the GLM coefficients (S4–S12 Tables) are provided as supporting information.

## Supporting Information

S1 Fig Linkage disequilibrium matrix. Linkage disequilibrium was assessed from a genomic region of 100 kb with *MtnA* at the center. The upper and right axes show the SNPs found in the 100 kb fragment, excluding singletons, and each cell represents *r*^2^ values. The left and bottom part of the matrix shows the results of a Fisher’s exact test for each pair of SNPs. Red boxes indicate significant *P*-values.
(PDF)

S2 Fig Nucleotide diversity (*π*) and site frequency spectrum (SFS) of chromosome arm 3R. (A) Nucleotide diversity (*π*) for 11 lines from the Netherlands (NL), eight lines from France (FR), all the Dutch and French lines combined (FR‐NL), and the French line FR14 combined with 11 lines from the Netherlands (FR14‐NL). (B) Dark blue bars indicate the SFS for the 11 Dutch lines for which complete genome sequences are available. Light blue bars indicate the SFS of 10 of these Netherlands lines plus one French line. In order to have a constant sample size of 12 for the *SweepFinder* analysis, one French line (FR14) was included with the NL lines to calculate the background site frequency spectrum.
(PDF)

S3 Fig Oxidative stress tolerance by sex. Proportional mortality of *D*. *melanogaster* males (A, C) and females (B, D) after exposure to hydrogen peroxide for 48 hours in (A,B) flies with (hatched lines) and without (solid lines) the deletion in the *MtnA* 3’ UTR and (C,D) RNAi-mediated *MtnA* knockdown (hatched lines) and control (solid lines) flies (C,D). (A,B) The Dutch (NL) population is shown in blue and the Malaysian (KL) population in orange. Legends are provided to the right of each row. *P*-values are shown for within population/background and sex comparisons. Error bars represent standard error of the mean.
(PDF)

S1 Table Isoform-specific expression of *MtnA* in the brain. (PDF)

S2 Table Average pairwise differences per kb between French (FR) and Dutch (NL) lines. (PDF)

S3 Table Site frequency spectrum (SFS) of the Swedish population drawn from the whole 3R chromosome arm. (PDF)

S4 Table Oxidative stress tolerance glm coefficients for Malaysian population. (PDF)

S5 Table Oxidative stress tolerance glm coefficients for Dutch population. (PDF)

S6 Table Oxidative stress tolerance glm coefficients for *MtnA* knockdown and control lines. (PDF)

S7 Table Male oxidative stress tolerance glm coefficients for Malaysian population. (PDF)

S8 Table Male oxidative stress tolerance glm coefficients for Dutch population. (PDF)

S9 Table Male oxidative stress tolerance glm coefficients for *MtnA* knockdown and control lines. (PDF)

S10 Table Female oxidative stress tolerance glm coefficients for Malaysian population. (PDF)

S11 Table Female oxidative stress tolerance glm coefficients for Dutch population. (PDF)

S12 Table Female oxidative stress tolerance glm coefficients for *MtnA* knockdown and control lines. (PDF)
