# Supplementary material for: An Indel Polymorphism in the MtnA 3' Untranslated Region Is Associated with Gene Expression Variation and Local Adaptation in Drosophila melanogaster
Source: PLoS Genet. 2016 Apr 27;12(4):e1005987. doi: 10.1371/journal.pgen.1005987 (PMC4847869; doi:10.1371/journal.pgen.1005987)
Supplement: S1 Fig — Linkage disequilibrium was assessed from a genomic region of 100 kb with MtnA at the center. The upper and right axes show the SNPs found in the 100 kb fragment, excluding singletons, and each cell represents r2 values. The left and bottom part of the matrix shows the results of a Fisher’s exact test for each pair of SNPs. Red boxes indicate significant P-values. (PDF) [file pgen.1005987.s001.pdf]

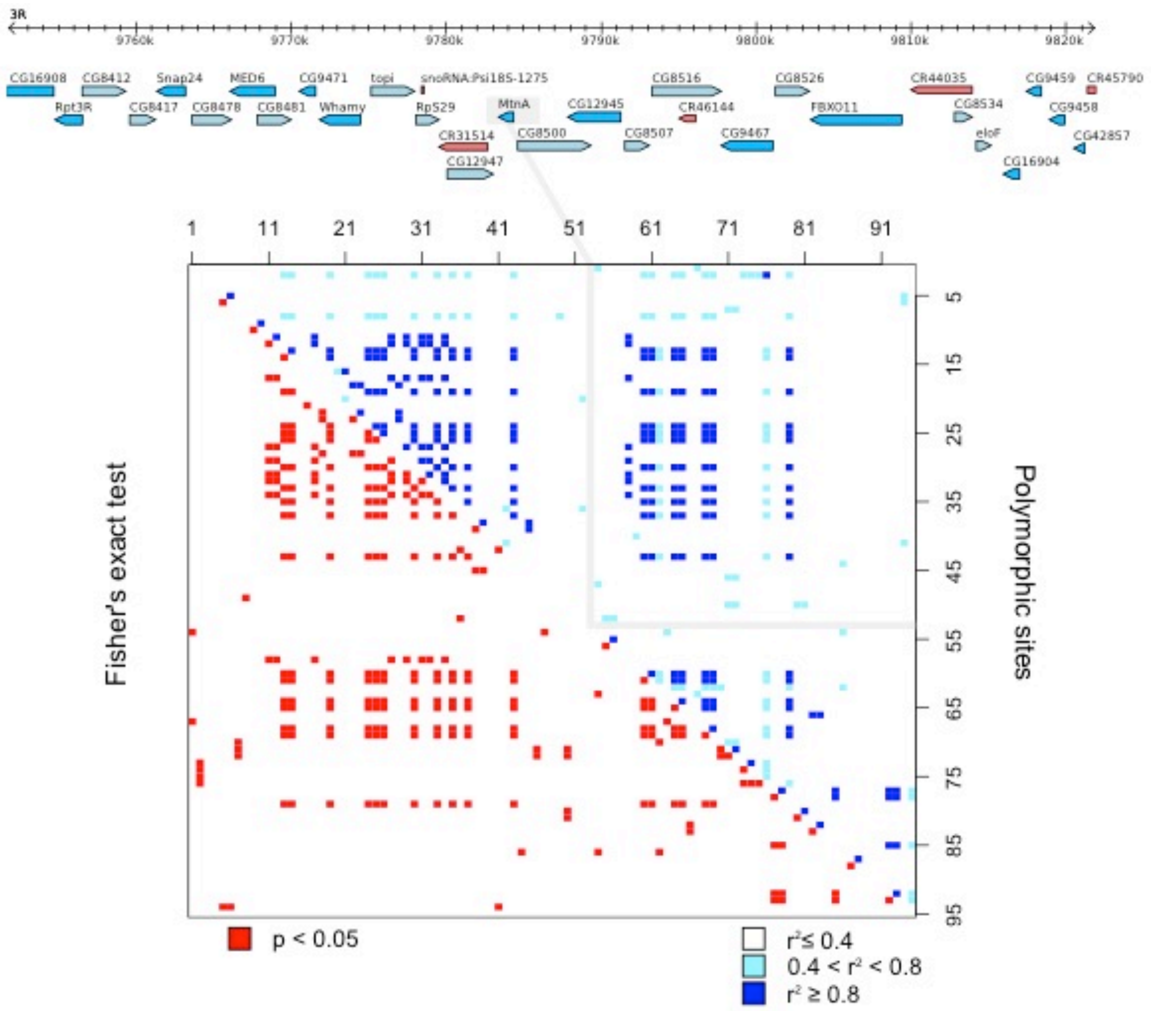

S1 Fig. Linkage disequilibrium matrix. Linkage disequilibrium was assed from a genomic region of 100 kb with *MtnA* at the center. The upper and right axes show the SNPs found in the 100 kb fragment not taking into account singletons and each bin represent r^2 values. The left and bottom part of the matrix show the Fisher exact test for each of the r^2 values. Red boxes indicate significant p-values.
