# Supplementary material for: An Indel Polymorphism in the MtnA 3' Untranslated Region Is Associated with Gene Expression Variation and Local Adaptation in Drosophila melanogaster
Source: PLoS Genet. 2016 Apr 27;12(4):e1005987. doi: 10.1371/journal.pgen.1005987 (PMC4847869; doi:10.1371/journal.pgen.1005987)
Supplement: S2 Fig — (A) Nucleotide diversity (π) for 11 lines from the Netherlands (NL), eight lines from France (FR), all the Dutch and French lines combined (FR‐NL), and the French line FR14 combined with 11 lines from the Netherlands (FR14‐NL). (B) Dark blue bars indicate the SFS for the 11 Dutch lines for which complete genome sequences are available. Light blue bars indicate the SFS of 10 of these Netherlands lines plus one French line. In order to have a constant sample size of 12 for the SweepFinder analysis, one French line (FR14) was included with the NL lines to calculate the background site frequency spectrum. (PDF) [file pgen.1005987.s002.pdf]

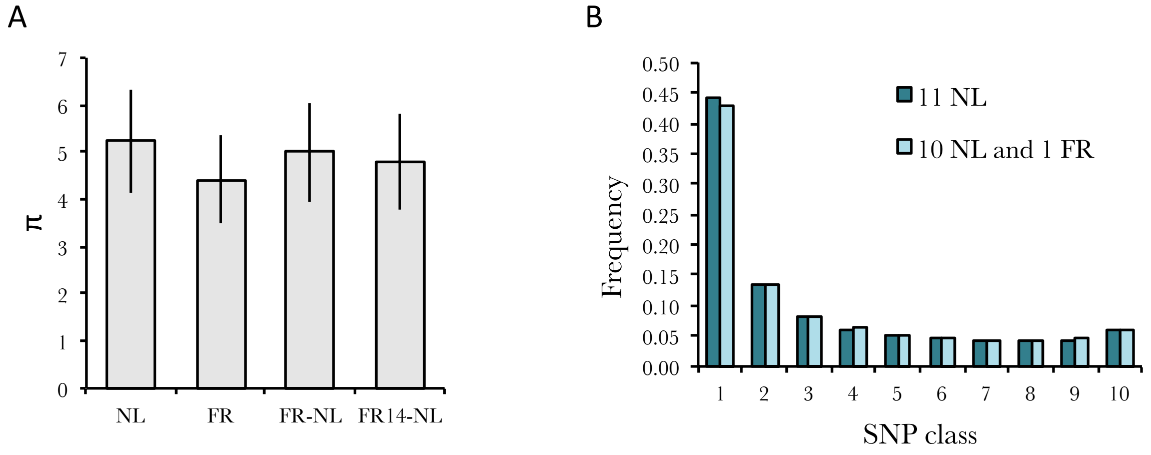

S2 Fig. Nucleotide diversity (*π*) and site frequency spectrum (SFS) of chromosome arm 3R. (A) Nucleotide diversity (*π*) for 11 lines from the Netherlands (NL), eight lines from France (FR), all the Dutch and French lines combined (FR-NL), and the French line FR14 combined with 11 lines from the Netherlands (FR14-NL). (B) Dark blue bars indicate the SFS for the 11 Dutch lines for which complete genome sequences are available. Light blue bars indicate the SFS of 10 of these Netherlands lines plus one French line. In order to have a constant sample size of 12 for the SweepFinder analysis, one French line (FR14) was included with the NL lines to calculate the background site frequency spectrum.
