# Supplementary material for: An Indel Polymorphism in the MtnA 3' Untranslated Region Is Associated with Gene Expression Variation and Local Adaptation in Drosophila melanogaster
Source: PLoS Genet. 2016 Apr 27;12(4):e1005987. doi: 10.1371/journal.pgen.1005987 (PMC4847869; doi:10.1371/journal.pgen.1005987)
Supplement: S3 Fig — Proportional mortality of D. melanogaster males (A, C) and females (B, D) after exposure to hydrogen peroxide for 48 hours in (A,B) flies with (hatched lines) and without (solid lines) the deletion in the MtnA 3’ UTR and (C,D) RNAi-mediated MtnA knockdown (hatched lines) and control (solid lines) flies (C,D). (A,B) The Dutch (NL) population is shown in blue and the Malaysian (KL) population in orange. Legends are provided to the right of each row. P-values are shown for within population/background and sex comparisons. Error bars represent standard error of the mean. (PDF) [file pgen.1005987.s003.pdf]

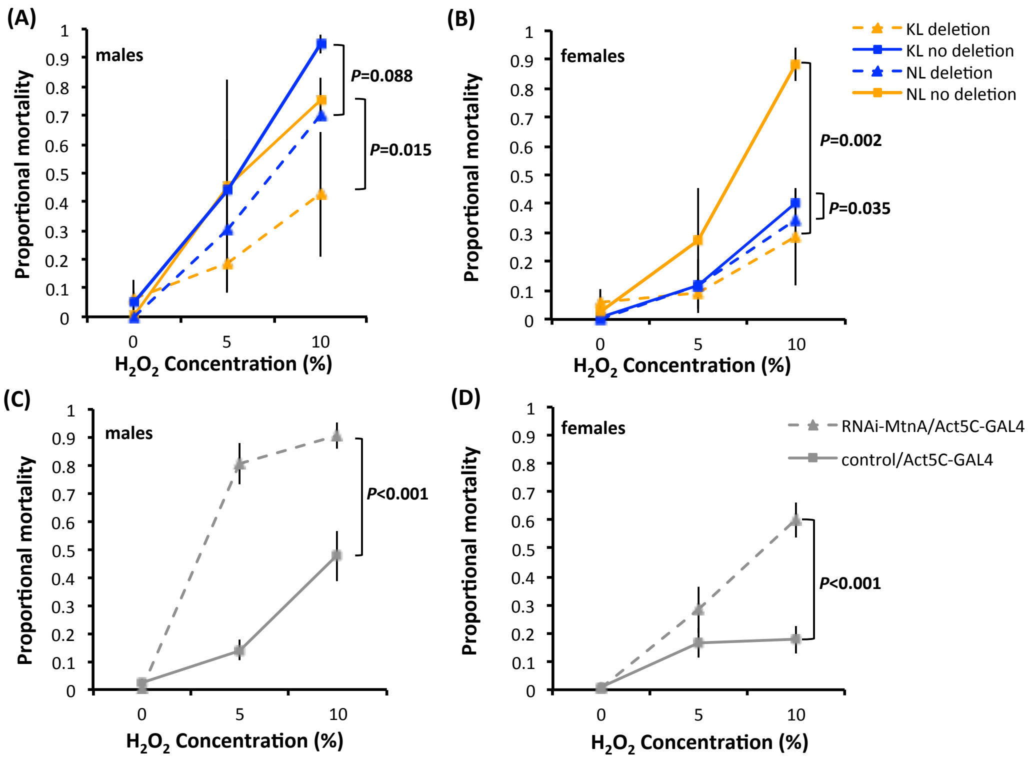

S3 Fig. Oxidative stress tolerance by sex. Proportional mortality of *D. melanogaster* males (A, C) and females (B, D) after exposure to hydrogen peroxide for 48 hours in (A,B) flies with (hatched lines) and without (solid lines) the deletion in the *MtnA* 3' UTR and (C,D) RNAi-mediated *MtnA* knockdown (hatched lines) and control (solid lines) flies (C,D). (A,B) The Dutch (NL) population is shown in blue and the Malaysian (KL) population in orange. Legends are provided to the right of each row. *P*-values are shown for within population/background and sex comparisons. Error bars represent standard error of the mean.
