# Supplementary material for: An Indel Polymorphism in the MtnA 3' Untranslated Region Is Associated with Gene Expression Variation and Local Adaptation in Drosophila melanogaster
Source: PLoS Genet. 2016 Apr 27;12(4):e1005987. doi: 10.1371/journal.pgen.1005987 (PMC4847869; doi:10.1371/journal.pgen.1005987)
Supplement: S1 Table — (PDF) [file pgen.1005987.s004.pdf]

S1 Table. Isoform-specific expression of *MtnA* in the brain.

	Expression (RPKM)		
**Population**	***MtnA* shared (329 bp)**	***MtnA-RB* specific (371 bp)**	**% *MtnA-RB***
The Netherlands	3867.74	57.94	1.50
Zimbabwe	859.74	1.12	0.13
