# Supplementary material for: An Indel Polymorphism in the MtnA 3' Untranslated Region Is Associated with Gene Expression Variation and Local Adaptation in Drosophila melanogaster
Source: PLoS Genet. 2016 Apr 27;12(4):e1005987. doi: 10.1371/journal.pgen.1005987 (PMC4847869; doi:10.1371/journal.pgen.1005987)
Supplement: S2 Table — (PDF) [file pgen.1005987.s005.pdf]

S2 Table. Average pairwise differences per kb between French (FR) and Dutch (NL) lines.

	FR151	FR180	FR207	FR217	FR229	FR310	FR361	NL11	NL12	NL13	NL14	NL15	NL16	NL17	NL18	NL19	NL1	NL2
FR14	4.1	5.6	4.1	6.1	1.8	4.3	5.9	5.2	4.4	6.1	4.2	4.9	3.3	4.7	4.8	5.3	4.7	5.1
FR151		5.7	4.1	6.3	2.1	4.4	5.9	5.4	4.5	6.1	4.2	5.1	3.4	4.8	4.8	5.3	4.8	5.1
FR180			5.6	6.2	2	5.7	4.7	6.5	5.8	5.1	5.7	6.3	4.7	6	6.1	6.6	6	6.3
FR207				6.2	1.7	4.4	6	5.3	4.5	6	4.3	5.1	3.4	4.7	4.8	5.2	4.7	5.1
FR217					2.2	6.3	6.2	7	6.4	6.2	6.1	6.7	5.2	6.5	6.7	7.1	6.6	6.9
FR229						2.2	2.5	2.4	2.1	2.1	1.2	2	1.1	2.1	2.1	2.2	2.3	2.1
FR310							6	5.3	4.7	6.2	4.5	5.3	3.6	4.8	3.1	5.4	4.9	5.3
FR361								6.8	6.1	5.1	5.9	6.6	5	6.3	6.4	6.9	6.3	6.6
NL11									5.4	7	5.2	6	4.4	5.6	5.8	6.2	5.7	6.1
NL12										6.3	4.3	5.2	3.5	4.8	5.1	5.4	4.9	5.1
NL13											6	6.8	5.2	6.5	6.5	7.1	6.5	6.8
NL14												4.6	3.2	4.5	4.9	4.9	4.6	4.9
NL15													2.1	5.3	5.7	5.8	5.1	5.7
NL16														3.5	4.1	4.4	3.7	4.1
NL17															5.3	5.7	5.3	5.2
NL18																5.8	5.4	5.7
NL19																	5.7	6
NL1																		3.7
