# Supplementary material for: An Indel Polymorphism in the MtnA 3' Untranslated Region Is Associated with Gene Expression Variation and Local Adaptation in Drosophila melanogaster
Source: PLoS Genet. 2016 Apr 27;12(4):e1005987. doi: 10.1371/journal.pgen.1005987 (PMC4847869; doi:10.1371/journal.pgen.1005987)
Supplement: S3 Table — (PDF) [file pgen.1005987.s006.pdf]

S3 Table. Site frequency spectrum (SFS) of the Swedish population drawn from the whole 3R chromosome arm.

Class	Frequency
1	0.799963
2	0.086063
3	0.025863
4	0.015205
5	0.011744
6	0.009859
7	0.008616
8	0.007853
9	0.007445
10	0.007420
11	0.008246
12	0.011711
