# Supplementary material for: An Indel Polymorphism in the MtnA 3' Untranslated Region Is Associated with Gene Expression Variation and Local Adaptation in Drosophila melanogaster
Source: PLoS Genet. 2016 Apr 27;12(4):e1005987. doi: 10.1371/journal.pgen.1005987 (PMC4847869; doi:10.1371/journal.pgen.1005987)
Supplement: S4 Table — (PDF) [file pgen.1005987.s007.pdf]

S4 Table. Oxidative stress tolerance glm coefficients for the Malaysian population

	**Estimate**	**Std. Error**	**t value**	***P*-value**
**Intercept**	4.16137	0.49944	8.332	6.13E-14
**Concentration**	-0.3314	0.04201	-7.888	7.43E-13
**Deletion present**	2.91704	0.88944	3.28	0.001307
**Line KL02**	-1.11336	0.40784	-2.73	0.007138
**Line KL10**	-3.71588	0.88483	-4.2	4.70E-05
**Line KL11**	-3.03958	0.89059	-3.413	0.000837
**sex male**	-1.18672	0.29168	-4.069	7.81E-05
