# Supplementary material for: An Indel Polymorphism in the MtnA 3' Untranslated Region Is Associated with Gene Expression Variation and Local Adaptation in Drosophila melanogaster
Source: PLoS Genet. 2016 Apr 27;12(4):e1005987. doi: 10.1371/journal.pgen.1005987 (PMC4847869; doi:10.1371/journal.pgen.1005987)
Supplement: S5 Table — (PDF) [file pgen.1005987.s008.pdf]

S5 Table. Oxidative stress tolerance glm coefficients for the Dutch population

	Estimate	Std. Error	t value	*P*-value
**Intercept**	3.38682	0.52533	6.447	6.86E-09
**Concentration**	-0.4679	0.06073	-7.705	2.36E-11
**Deletion present**	1.5757	0.44937	3.506	0.000732
**Line NL17**	-0.0708	0.43904	-0.161	0.872275
**sex male**	-1.15475	0.36258	-3.185	2.03E-03
