# Supplementary material for: An Indel Polymorphism in the MtnA 3' Untranslated Region Is Associated with Gene Expression Variation and Local Adaptation in Drosophila melanogaster
Source: PLoS Genet. 2016 Apr 27;12(4):e1005987. doi: 10.1371/journal.pgen.1005987 (PMC4847869; doi:10.1371/journal.pgen.1005987)
Supplement: S6 Table — (PDF) [file pgen.1005987.s009.pdf]

S6 Table. Oxidative stress tolerance glm coefficients for *MtnA*knockdown and control lines

	Estimate	Std. Error	t value	*P*-value
**Intercept**	-5.55789	0.53808	-10.329	2.00E-16
**Concentration**	0.42612	0.04458	9.559	4.99E-15
**Line**	2.08414	0.33802	6.166 2	4.30E-09
**sex male**	1.54003	0.30396	5.067	2.41E-06
