# Supplementary material for: An Indel Polymorphism in the MtnA 3' Untranslated Region Is Associated with Gene Expression Variation and Local Adaptation in Drosophila melanogaster
Source: PLoS Genet. 2016 Apr 27;12(4):e1005987. doi: 10.1371/journal.pgen.1005987 (PMC4847869; doi:10.1371/journal.pgen.1005987)
Supplement: S7 Table — (PDF) [file pgen.1005987.s010.pdf]

S7 Table. Male oxidative stress tolerance glm coefficients for the Malaysian population

	**Estimate**	**Std. Error**	**t value**	***P*-value**
**Intercept**	3.4931	0.6327	5.521	5.72E-07
**Concentration**	-0.3786	0.0614	-6.166	4.35E-08
**Deletion present**	3.0115	1.2059	2.497	0.01494
**Line KL02**	-1.8723	0.6116	-3.062	0.00315
**Line KL10**	-3.8161	1.2074	-3.161	2.35E-03
**Line KL11**	-3.2141	1.208	-2.661	0.00972
