# Supplementary material for: An Indel Polymorphism in the MtnA 3' Untranslated Region Is Associated with Gene Expression Variation and Local Adaptation in Drosophila melanogaster
Source: PLoS Genet. 2016 Apr 27;12(4):e1005987. doi: 10.1371/journal.pgen.1005987 (PMC4847869; doi:10.1371/journal.pgen.1005987)
Supplement: S8 Table — (PDF) [file pgen.1005987.s011.pdf]

S8 Table. Male oxidative stress tolerance glm coefficients for the Dutch population

	Estimate	Std. Error	t value	*P*-value
**Intercept**	2.537163	0.62857	4.036	2.31E-04
**Concentration**	-0.477495	0.085953	-5.555	1.85E-06
**Deletion present**	1.162638	0.664607	1.749	0.087711
**Line NL17**	0.003915	0.649791	0.006	0.995222
