# Supplementary material for: An Indel Polymorphism in the MtnA 3' Untranslated Region Is Associated with Gene Expression Variation and Local Adaptation in Drosophila melanogaster
Source: PLoS Genet. 2016 Apr 27;12(4):e1005987. doi: 10.1371/journal.pgen.1005987 (PMC4847869; doi:10.1371/journal.pgen.1005987)
Supplement: S9 Table — (PDF) [file pgen.1005987.s012.pdf]

S9 Table. Male oxidative stress tolerance glm coefficients for *MtnA* knockdown and control lines

	Estimate	Std. Error	t value	*P*-value
**Intercept**	-5.1622	0.8194	-6.3	2.21E-07
**Concentration**	0.5323	0.0824	6.46	1.34E-07
**Line**	2.9362	0.6192	4.742	2.96E-05
