# Supplementary material for: An Indel Polymorphism in the MtnA 3' Untranslated Region Is Associated with Gene Expression Variation and Local Adaptation in Drosophila melanogaster
Source: PLoS Genet. 2016 Apr 27;12(4):e1005987. doi: 10.1371/journal.pgen.1005987 (PMC4847869; doi:10.1371/journal.pgen.1005987)
Supplement: S10 Table — (PDF) [file pgen.1005987.s013.pdf]

S10 Table. Female oxidative stress tolerance glm coefficients for the Malaysian population

	Estimate	Std. Error	t value	*P*-value
**Intercept**	3.59488	0.61971	5.801	1.82E-07
**Concentration**	-0.28988	0.05879	-4.931	5.42E-06
**Deletion present**	2.73793	1.27415	2.149	0.03516
**Line KL02**	-0.30477	0.56493	-0.539	0.59129
**Line KL10**	-3.51212	1.25831	-2.791	6.79E-03
**Line KL11**	-2.7662	1.27759	-2.165	0.03384
