# Supplementary material for: An Indel Polymorphism in the MtnA 3' Untranslated Region Is Associated with Gene Expression Variation and Local Adaptation in Drosophila melanogaster
Source: PLoS Genet. 2016 Apr 27;12(4):e1005987. doi: 10.1371/journal.pgen.1005987 (PMC4847869; doi:10.1371/journal.pgen.1005987)
Supplement: S11 Table — (PDF) [file pgen.1005987.s014.pdf]

S11 Table. Female oxidative stress tolerance glm coefficients for the Dutch population

	Estimate	Std. Error	t value	*P*-value
**Intercept**	3.00525	0.68272	4.402	7.78E-05
**Concentration**	-0.45054	0.08932	-5.044	1.03E-05
**Deletion present**	2.03235	0.62754	3.239	0.00242
**Line NL17**	-0.16871	0.62394	-0.27	0.78824
