# Supplementary material for: An Indel Polymorphism in the MtnA 3' Untranslated Region Is Associated with Gene Expression Variation and Local Adaptation in Drosophila melanogaster
Source: PLoS Genet. 2016 Apr 27;12(4):e1005987. doi: 10.1371/journal.pgen.1005987 (PMC4847869; doi:10.1371/journal.pgen.1005987)
Supplement: S12 Table — (PDF) [file pgen.1005987.s015.pdf]

S12 Table. Female oxidative stress tolerance glm coefficients for *MtnA* knockdown and control lines

	Estimate	Std. Error	t value	*P*-value
**Intercept**	-4.56657	0.52753	-8.657	5.69E-11
**Concentration**	0.35188	0.04982	7.063	1.04E-08
**Line**	1.46324	0.38049	3.846	3.92E-04
